# Participatory women’s groups and counselling through home visits to improve child growth in rural eastern India: protocol for a cluster randomised controlled trial

**DOI:** 10.1186/s12889-015-1655-z

**Published:** 2015-04-15

**Authors:** Nirmala Nair, Prasanta Tripathy, Harshpal S Sachdev, Sanghita Bhattacharyya, Rajkumar Gope, Sumitra Gagrai, Shibanand Rath, Suchitra Rath, Rajesh Sinha, Swati Sarbani Roy, Suhas Shewale, Vijay Singh, Aradhana Srivastava, Hemanta Pradhan, Anthony Costello, Andrew Copas, Jolene Skordis-Worrall, Hassan Haghparast-Bidgoli, Naomi Saville, Audrey Prost

**Affiliations:** Ekjut, Chakradharpur, Jharkhand India; Sitaram Bhartia Institute of Science and Research, New Delhi, India; Public Health Foundation of India, New Delhi, India; Institute for Global Health, University College London, 30 Guilford Street, London, WC1N 1EH United Kingdom; MRC Clinical Trials Unit at University College London, London, UK

**Keywords:** Stunting, India, Child, Nutrition, Public health

## Abstract

**Background:**

Child stunting (low height-for-age) is a marker of chronic undernutrition and predicts children’s subsequent physical and cognitive development. Around one third of the world’s stunted children live in India. Our study aims to assess the impact, cost-effectiveness, and scalability of a community intervention with a government-proposed community-based worker to improve growth in children under two in rural India.

**Methods:**

The study is a cluster randomised controlled trial in two rural districts of Jharkhand and Odisha (eastern India). The intervention tested involves a community-based worker carrying out two activities: (a) one home visit to all pregnant women in the third trimester, followed by subsequent monthly home visits to all infants aged 0–24 months to support appropriate feeding, infection control, and care-giving; (b) a monthly women’s group meeting using participatory learning and action to catalyse individual and community action for maternal and child health and nutrition. Both intervention and control clusters also receive an intervention to strengthen Village Health Sanitation and Nutrition Committees.

The unit of randomisation is a purposively selected cluster of approximately 1000 population. A total of 120 geographical clusters covering an estimated population of 121,531 were randomised to two trial arms: 60 clusters in the intervention arm receive home visits, group meetings, and support to Village Health Sanitation and Nutrition Committees; 60 clusters in the control arm receive support to Committees only. The study participants are pregnant women identified in the third trimester of pregnancy and their children (n = 2520). Mothers and their children are followed up at seven time points: during pregnancy, within 72 hours of delivery, and at 3, 6, 9, 12 and 18 months after birth. The trial’s primary outcome is children’s mean length-for-age Z scores at 18 months. Secondary outcomes include wasting and underweight at all time points, birth weight, growth velocity, feeding, infection control, and care-giving practices. Additional qualitative and quantitative data are collected for process and economic evaluations.

**Discussion:**

This trial will contribute to evidence on effective strategies to improve children's growth in India.

**Trial registration:**

ISRCTN register 51505201; Clinical Trials Registry of India number 2014/06/004664.

**Electronic supplementary material:**

The online version of this article (doi:10.1186/s12889-015-1655-z) contains supplementary material, which is available to authorized users.

## Background

Undernutrition affects an estimated 165 million children in low and middle-income countries, and contributes to up to 45% of child deaths [1]. Stunting, or low length/height-for-age, is a marker of chronic undernutrition, and predicts subsequent physical and cognitive development [2,3]. Stunted children are more likely to do poorly in school and subsequently have low incomes, contributing to the intergenerational transmission of poverty [3]. The World Health Organisation has called for global action to reduce the proportion of children who are stunted by 40% by 2025 [4], and nutrition is now recognized as central to sustainable development [5].

**Figure 1:**
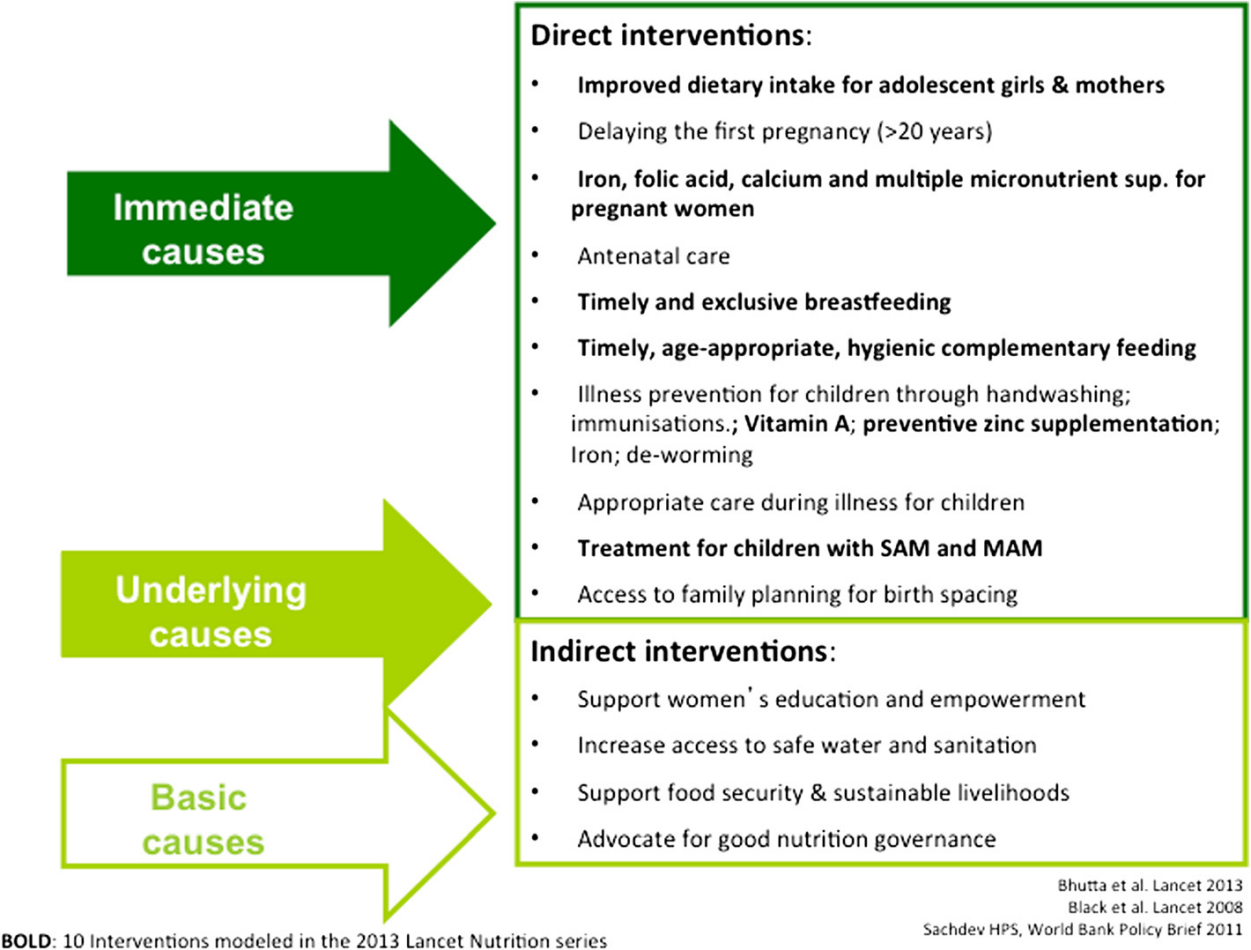
Interventions to address maternal and child undernutrition.

There is substantial scientific consensus on determinants of maternal and child undernutrition, and emerging agreement on interventions needed to reduce them. These are summarised in Figure 1. Current community strategies to prevent childhood stunting focus on intensifying the coverage of key interventions in pregnancy and the first two years of children’s lives, or the ‘1000 days’ period [6]. At the same time, there is recognition that substantial impact requires preventive action beyond the first 1000 days of life and outwith immediate health and food interventions. Such action should support adequate nutrition in early adolescence, prevent early pregnancies, improve access to clean water and sanitation to reduce environmental gastroenteropathy, support food security, and promote women’s empowerment [7-11]. A key challenge for community interventions in areas with a high burden of stunting therefore lies in seeking impact on its more immediate determinants (infection control, care practices and feeding in the first 1000 days of life), while supporting longer-term changes in more distal determinants.

Around one third of the world’s stunted children live in India, with the highest prevalence found in the poorest wealth quintiles and among Scheduled Tribe (ST) and Scheduled Caste (SC) communities [12,13]. Around 22% of growth faltering in young Indian children has already happened at birth, and the remainder is largely completed by 24 months [14,15]. The Government of India’s Integrated Child Development Scheme (ICDS) and National Rural Health Mission (NRHM) support many of the direct interventions in Figure 1, but the impact of these programmes has so far been limited: ICDS’ key cadre, the *Anganwadi Sevika*, spends most of her time record-keeping and providing food supplementation to children aged 3–6 years, and comparatively little time promoting growth among children under two [16]. Previous trials of community interventions to reduce undernutrition have either been conducted outside India [17], tested single interventions rather than integrated strategies [18-20], failed to focus on the critical window for growth or, except for one [21], failed to impact on children’s length/height [22-25]. India’s policy advisers have called for urgent efforts to reduce child undernutrition through changes to the ICDS and innovative interventions in 200 high-burden districts [26]. One proposed reform consists of recruiting a second *Anganwadi Sevika* to promote growth in infants and young children [27-29]. There is currently no evidence-based, rigorously evaluated, scalable intervention model for such a worker.

### Study aim

We aim to determine whether a strategy involving a community-based female worker (*Suposhan Karyakarta*, or SPK), modelled on a second *Anganwadi Sevika*, can reduce stunting in children under two by improving feeding, infection control, and care-giving practices.

## Methods

### Setting

The study takes place in two districts: West Singhbhum (Jharkhand) and Kendujhar (Odisha). These districts have a combined population of 3.3 million (>80% living in rural areas), and fewer than half of women are literate [30]. In both districts, over 90% of households have no access to a toilet facility [Ibid.]. The main sources of livelihood in the study areas include rice and millet cultivation, collection and sale of forest products, daily wage labour, and work in brick kilns or mines. Over 40% of community members in the districts are from indigenous communities (*adivasi*). *Adivasi* children have very high undernutrition rates: more than 50% are underweight and stunted, and over 75% are anaemic [31]. Villages in the study districts have a triad of frontline health and nutrition workers: an *Anganwadi Sevika*, who provides supplementary nutrition to pregnant women, breastfeeding mothers and children aged three to six years; an Accredited Social Health Activist (ASHA) who helps community members access essential health services and promotes institutional deliveries; and an Auxiliary Nurse Midwife (ANM), who provides immunisations, essential medicines and antenatal care.

Ekjut (http://www.ekjutindia.org/about-us.html), the civil society organisation leading the study, has been working in West Singhbhum and Kendujhar since 2004, with a focus on improving the health of indigenous communities through participatory approaches [32].

### Trial design

The study is a cluster randomised controlled trial in which 60 clusters are allocated to receive the intervention and 60 clusters act as controls.

### Cluster size and selection

The unit of randomisation is a purposively selected cluster of around 1000 population, consisting of a village and its adjoining hamlets. Clusters are separated by natural boundaries (rivers, hills, or distance) to avoid proximity between intervention and control areas.

### Randomisation

**Figure 2:**
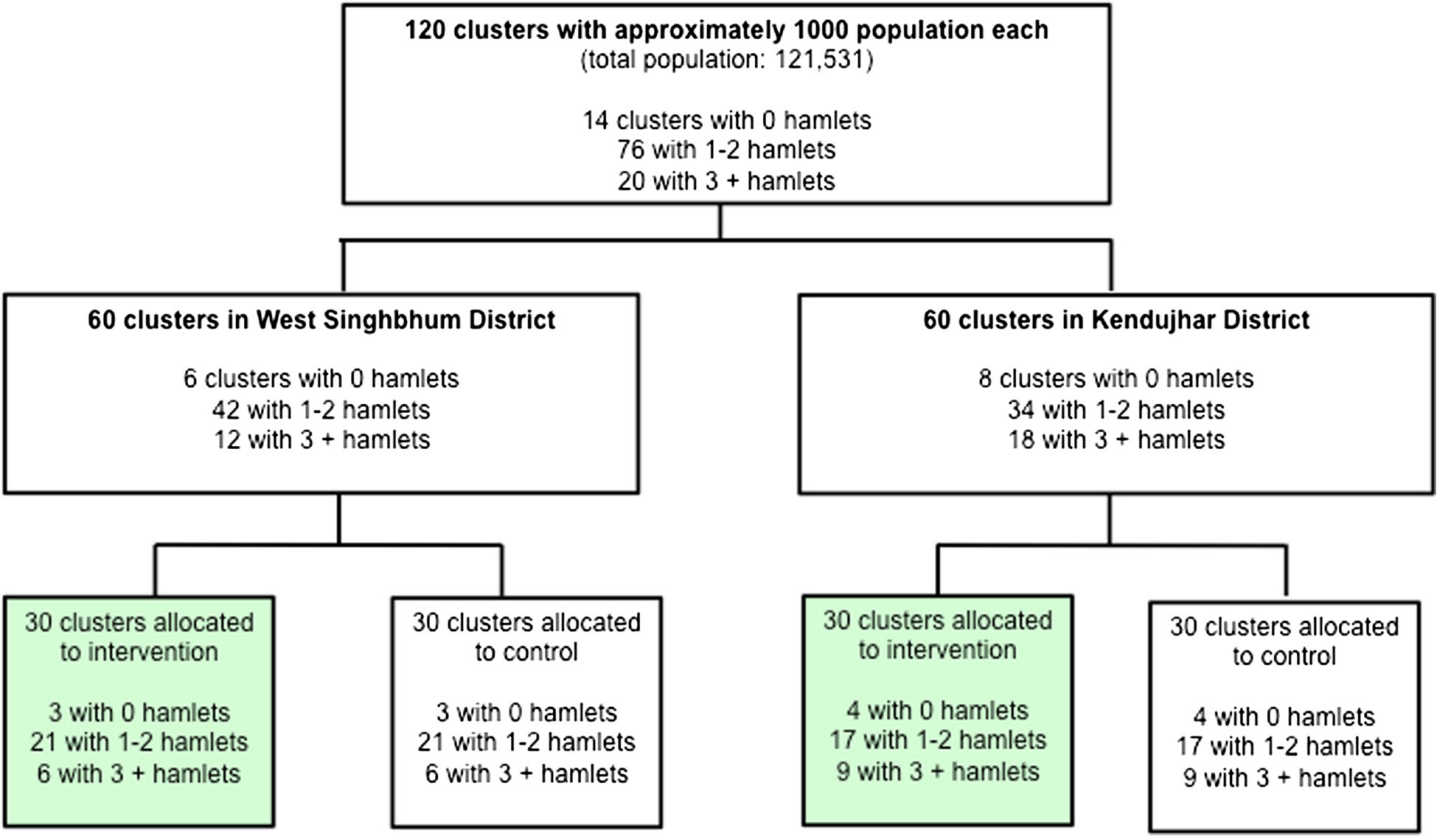
Trial design.

The randomisation, which took place in July 2013, was stratified by district and by number of hamlets per unit (see Figure 2: Trial design). In each district, Ekjut invited village leaders, teachers, frontline workers such as *Anganwadi Sevikas* and ASHAs, members of local governance bodies (Panchayati Raj Institutions), and Women and Child Development functionaries to participate in a meeting. Members of the research team explained the purpose of the study and the randomisation process. A list of numbered clusters was placed on a wall. The list was stratified into three groups: clusters with no hamlets, clusters with one to two hamlets, and clusters with three or more hamlets. Meeting participants placed a set of numbered balls corresponding to the clusters in each stratum in a local ‘tombola’ (lottery device), and sequentially allocated each ball/cluster to the intervention or control arms.

### Trial participants

Participants are women in the third trimester of pregnancy residing in the study clusters and identified between the 1st of October 2013 and 31st December 2015. Incentivised volunteers report every newly identified pregnancy to a member of the monitoring team. A study monitor then approaches the pregnant woman to explain the study and seek her consent for participation. Every pregnant woman residing in the study clusters and identified between the 1st October 2013 and 10th February 2015 is also asked for permission to follow-up her liveborn child for a period of 18 months. Pregnant women will be enrolled until the 31st December 2015 in order to measure the impact of the interventions on maternal nutrition in pregnancy.

We exclude stillbirths and neonatal deaths, infants whose mothers have died, infants with congenital abnormalities, multiple births, and mother/infant pairs who migrate out of the study area permanently during the study period. We define permanent migration as mother/infant pair missing more than two consecutive interviews (i.e. being absent from the study area for at least six months) at any point during the infant’s 18 months follow-up period.

### Intervention strategy

The intervention strategy involves a community-based worker (the SPK) carrying out home visits with individual families and participatory meetings with groups of women, to improve health and nutrition in the first 1000 days of life. The SPK is responsible for two main activities: (a) conducting a single home visit to each pregnant woman in the third trimester of pregnancy with counselling on maternal nutrition, followed by monthly home visits to all children under two, with counselling for growth promotion; (b) a monthly participatory meeting with a local women’s group. The SPK uses a problem-solving approach in both of these activities: during home visits, she identifies each mother’s current concerns about illness, feeding and care, then discusses possible solutions and barriers to putting the solutions into practice. During participatory meetings, she helps groups to identify community level health and nutrition problems, and to find locally feasible strategies to address them. We developed materials for these activities using formative research data from a quantitative nutrition survey conducted in 2010 in the study districts, qualitative discussions with women’s group members and facilitators participating in previous community interventions, and early intervention piloting data.

**Figure 3:**
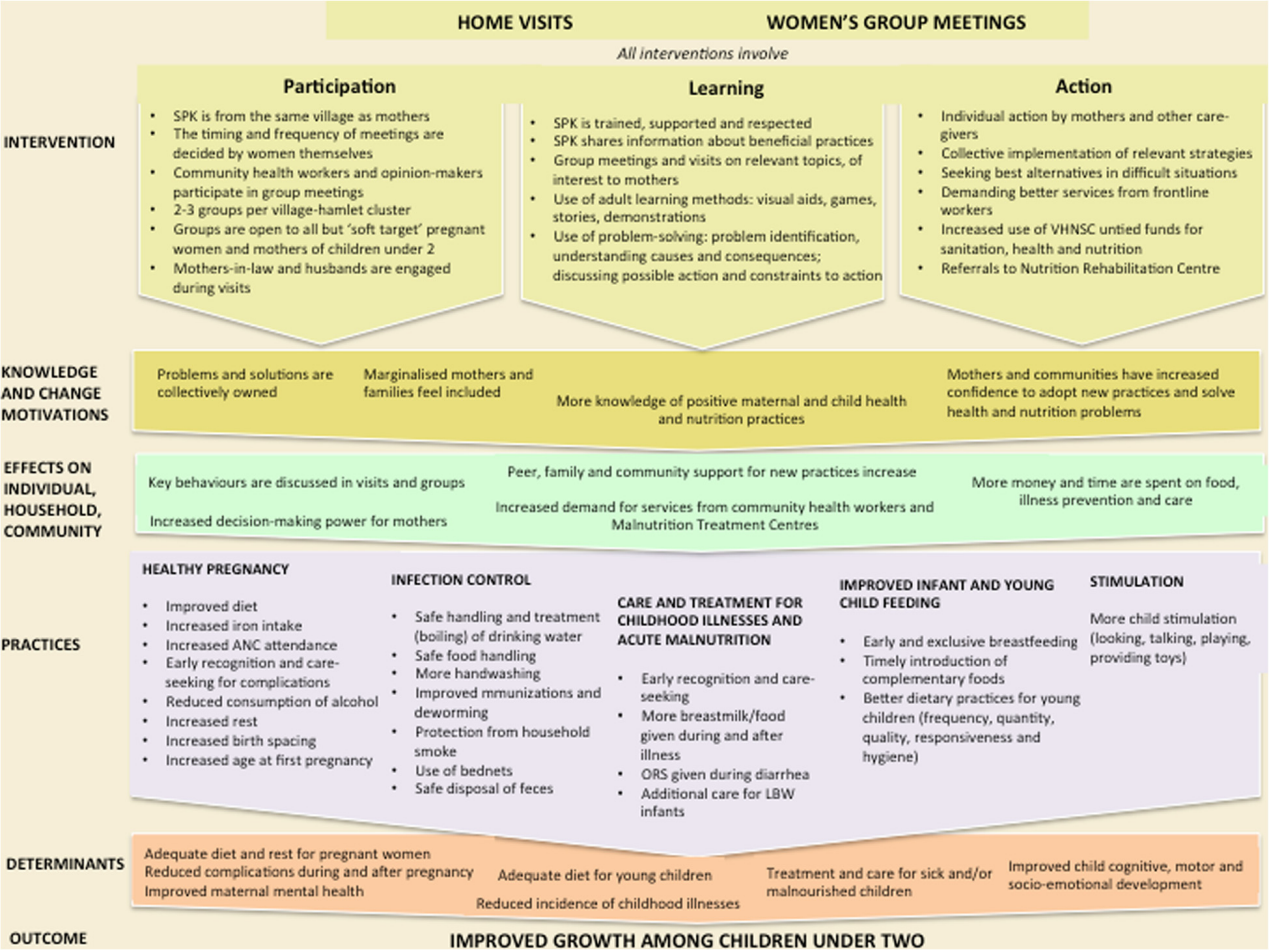
Theory of change.

**Figure 4:**
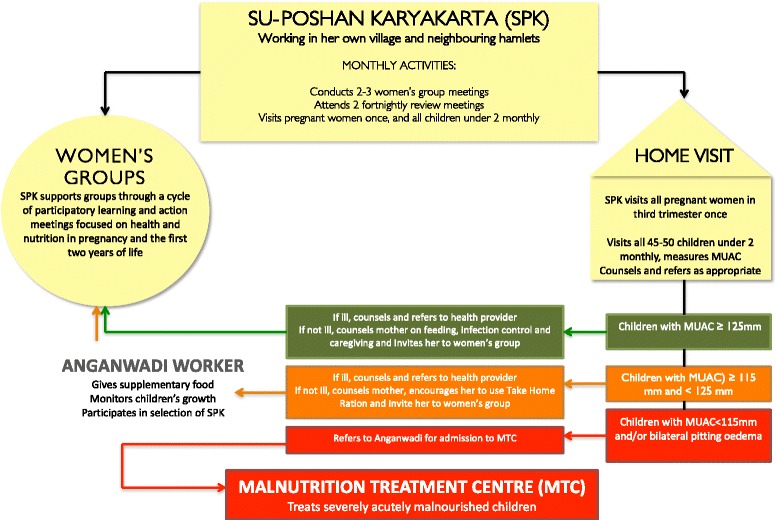
A village-level view of the community intervention strategy.

We propose this strategy because the current evidence suggests that an integrated approach to prevent undernutrition is more likely to succeed than discrete interventions (e.g. handwashing promotion or micronutrient supplementation on their own) [33-40]. We hypothesise that, to maximise its benefits, the strategy should include ‘fast-acting’ components to address immediate determinants of undernutrition (e.g. enhanced prevention, detection and treatment for infections, improved feeding and care-giving practices), as well as ‘slow-acting’ components to influence underlying determinants (e.g. unsafe water and sanitation, access to family planning, women and girls’ empowerment). The intervention’s Theory of Change is described in Figure 3. A detailed description of intervention activities is given below, and a village-level view of activities is shown in Figure 4.

#### Home visits

The SPK obtains the names and location of all pregnant women and children under two in her working area from the Anganwadi worker, women’s group members, and other community members. She visits all women at least once during pregnancy, and then visits all mothers of children under two on a monthly basis. At each visit to a mother and child, she asks about current or recent illness, takes a mid-upper arm circumference (MUAC) measurement for children over 6 months, then engages the mother in a dialogue about feeding, infection control, and care-giving practices. This dialogue starts with the mothers’ immediate concerns with illness, feeding and care, and then builds on the SPK’s own observations of the mothers’ behaviour with her child and of the household (e.g. water use, mosquito net availability) to further guide the counselling session. The SPK is aided by picture cards depicting good practices and containing prompts for recommendations tailored to the local context. Depending on the child’s age and MUAC status, the SPK can take one of four approaches:

1. Child visited is younger than 6 months
  The SPK responds to the mothers’ immediate concerns, provides appropriate breastfeeding counselling and, if the infant is ill, arranges to visit the Auxiliary Nurse Midwife or nearest appropriate health facility.
2. Child visited is older than 6 months, ill, and with green or yellow MUAC (≥115 mm)
  If the child is ill, the SPK gives recommendations on feeding during illness, checks the Mother Child Protection card – which contains records of immunisations - and asks about deworming, iron and vitamin A supplementation, Then, together with the mother, she makes a plan to visit the Auxiliary Nurse Midwife, Anganwadi, or the most appropriate local health facility.
3. Child visited is older than 6 months, not ill, with green or yellow MUAC (≥115 mm)
  The SPK asks about current child feeding practices, including what kinds of foods are given, in which frequency and quantity, then discusses how to use locally available foods in specific recipes to increase the nutrient and energy density of complementary foods, and demonstrating these in the mother’s home. The SPK then discusses the importance of handwashing and offers advice to improve the hygiene of food preparation and feeding. She also discusses and demonstrates childhood stimulation techniques with the mother or family.
4. Severely malnourished (MUAC <115 mm and/or bilateral pitting oedema)
  The SPK refers all severely malnourished children to the *Anganwadi* and assists with onward referral to Nutrition Rehabilitation Centre (NRC). She visits children with Severe Acute Malnutrition (SAM) following their discharge from the MTC and counsels the mother on feeding the child for recovery.

We chose to use MUAC as a screening tool because monthly weight measurement is collected by the existing *Anganwadi*. In addition, national and international evidence remains unclear about the actual benefit of plotting weight for growth promotion, and a critical review found that measuring and recording left little time for counselling and problem-solving with mothers [41]. Community-based management of acute malnutrition programmes commonly use MUAC to screen children over six months for moderate and acute malnutrition. This method is easily accepted by community health workers, and MUAC < 115 mm is a better predictor of mortality risk in malnourished children than weight-for-height < −3 [42].

We chose a guided but flexible approach to counselling for two reasons. Half of children under two in the study districts are underweight and over 60% are stunted [13]. Doing triage based on wasting or underweight to identify which prevention and treatment measures to discuss during home visits seemed superfluous, as most mothers would require counselling. Using strict algorithms for counselling also implies a strong reliance on job aides and standardized messages. While these are helpful and used to some extent in this intervention, we felt that it would be more empowering to give the SPK a basic sequence for the session (i.e. discussing illness, feeding, infection control and care-giving), along with picture cards containing key messages, but also give them the freedom to use their judgment and local knowledge to adapt the session to each mother.

#### Participatory meetings with women’s groups

**Figure 5:**
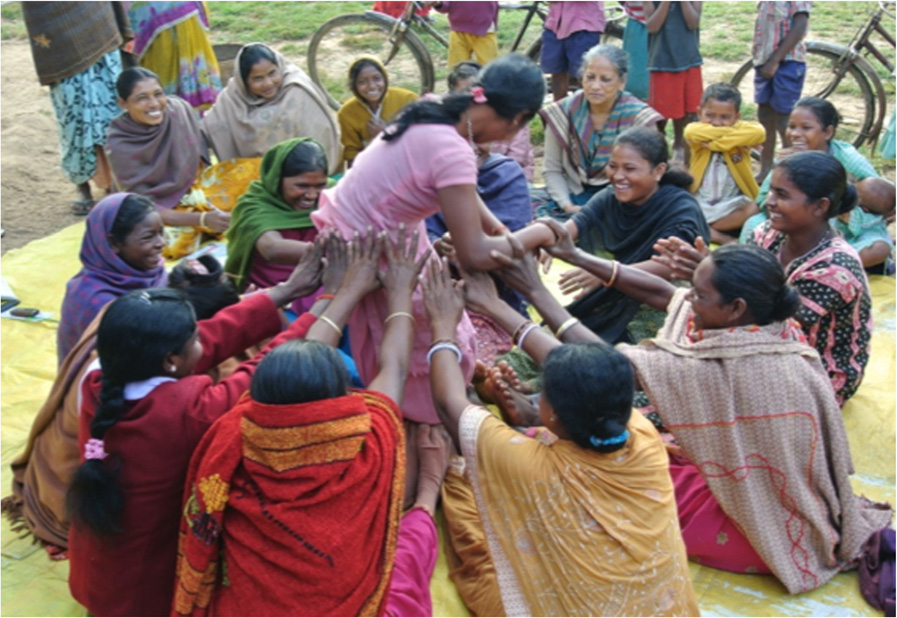
Women and girls discuss the benefits of working as a group.

The SPK also carries out a cycle of 20 monthly participatory meetings with groups of women, focusing on maternal and child health and nutrition. These meetings primarily target pregnant and lactating mothers, mothers of children under two, and adolescent girls (see Figure 5). They are however open to other community members because our previous experience with groups shows that this is important for inclusion and dissemination of information. The groups follow a four-phase participatory learning and action cycle to: (1) assess the health and nutrition situation; (2) decide on actions to take; (3) take action; (4) evaluate the process. In the first phase, the SPK introduces groups to the meeting cycle. Groups play a participatory game to discuss which mothers do not receive health and nutrition entitlements, discuss reasons for this and invite those mothers to join the meetings if they are not already attending. In the next meeting, the SPK describes the intergenerational cycle of undernutrition using a pictorial diagram and encourages the group to discuss local views and practices relating to undernutrition. Groups play a picture card game to identify health, feeding and caregiving problems related to undernutrition. The problems highlighted in the game are pre-identified by the study team on the basis of formative research. Group participants are invited to prioritise the problems that they would like to address by voting using the picture cards. In the second phase, groups explore the causes of their prioritised problems. They do this by listening to stories created by the SPKs using local themes. The stories illustrate how health and nutrition problems are linked to underlying household, community-level and health-service related issues. Group members discuss these underlying causes, then decide which of them they want to address and how. They allocate responsibilities for each strategy, decide on indicators to measure progress, and plan a community meeting to share their strategies with other community members and enlist their support. In the third phase, group members implement and review the strategies they have decided upon. At this time the SPK also introduces group members to other positive strategies for them to try at home, including how to enhance the density and diversity of complementary foods using local products and practice responsive feeding. In the fourth and final phase, group members discuss the progress of their strategies and share achievements and difficulties encountered during the meeting cycle. It is anticipated that the SPKs will coordinate at a full meeting cycle in the course of the trial, resulting in a range of individual, household and community-level strategies.

##### Recruitment, supervision and training of intervention workers

In the intervention arm, we recruited 60 SPKs through consultation with local Village Health Sanitation and Nutrition Committees (VHSNCs) and *Anganwadi Sevikas*. The SPK’s working area is her own village and its attached hamlets. She is paid a monthly stipend equivalent to that of existing *Anganwadi Sevikas.*

Six supervisors recruited by the study team support 10 SPKs each. SPKs and their supervisors receive phased trainings and attend bi-monthly supervision meetings. For home visits, training sessions covered knowledge and skills for counselling to improve Infant and Young Child Feeding practices, care-seeking for common childhood illnesses, and household-based strategies to prevent illness including handwashing, safe handling of water and food, and disposal of faeces. The training preparing SPKs for participatory group meetings included practical sessions on conducting women’s group meetings using a participatory learning and action approach, as well as interacting with existing community health workers.

##### Strengthening Village Health Sanitation and Nutrition Committees (VHSNCs)

In both intervention and controls arms, we will seek to strengthen the capacity of VHSNCs, which are village-level, government-mandated bodies responsible for assessing community health needs, preparing and implementing village health plans, and monitoring the provision of local health and nutrition services under NRHM. We will hold eight quarterly participatory meetings with VHSNCs over a period of two years. These meetings will involve participatory discussions to sensitise members to issues related to inequity and exclusion from health and nutrition services and entitlements, as well as identifying and addressing gaps in health and nutrition services. Ekjut have developed meeting plans and tools for engaging with Village Health Committees in Jharkhand, Odisha, and Madhya Pradesh. We propose to undertake this activity as a minimum common service to intervention and control areas because it has potential for long-term benefit and the work of VHSNCs can be sustained beyond the trial period at no added cost to local health services.

### Research questions

#### Primary research question

What is the impact and cost-effectiveness of a community strategy with participatory women’s groups and home visits, on linear growth among children 0–18 months in rural India?

#### Secondary research questions

1. Does the strategy lead to improvements in feeding, infection control and caring practices for children 0–18 months?
2. Does the strategy lead to increases in food intake and dietary diversity for women during pregnancy and lactation?
3. What operational factors affect the strategy’s delivery and impact?
4. What are the potential scalability, operational requirements, cost and impact of the strategy beyond the trial area?

### Sample size

The primary outcome of the study is mean length for age Z (LAZ) score at 18 months. In a meta-analysis of complementary feeding trials testing interventions to improve growth, nutrition education interventions had a mean effect size of 0.2 Z scores on LAZ at 18 months (range 0.04, 0.64) [38]. We therefore chose 0.15 as a conservative estimate of the effect size. The intra-cluster correlation (ICC) for stunting was estimated at 0.035 using an analysis of 42 DHS studies and our own data from the study areas [43]. The unit of randomisation (cluster) is a village and its attached hamlets. We expect around 25 live births per year in each cluster. Accounting for 10% attrition due to loss to follow-up and mortality, recruiting all eligible children born over 12 months in 110 clusters (2772 children before and 2520 after attrition), will allow us to detect a difference of 0.15 in mean LAZ scores between intervention and control arms (SD of 1) with 80% power and alpha of 0.05, and a design effect (D) of 1.8, calculated as D = 1 + (25–1) x 0.035. This sample size also gives the trial 80% power to detect a 13% reduction in the prevalence of stunting (from 60% to 52.5%), a 19% reduction in the prevalence of wasting (from 40% to 32.5%), and a reduction in the infant mortality rate greater than 45% (from 100 to 55 per 1000 livebirths) [44]. To account for the possibility of clusters dropping out of the study and for attrition rates exceeding our 10% estimate, we enrolled a total of 120 clusters (villages).

### Data collection, cleaning and storage

Across the study area, incentivised volunteers report pregnancies to monitors, each responsible for a population of around 4000. A monitor visits every pregnant woman identified, ascertains the estimated month of pregnancy, offers information about the study and invites her to join. If consent is given, the monitor measure the woman’s height and MUAC, and administers a first questionnaire. All children born to pregnant women recruited between 1st October 2013 and 10th February 2015 will be followed up within 72 hours of birth, and at 3, 6, 9, 12 and 18 months. Trained growth monitors conduct measurements at birth, and then visit the mother and child at 3, 6, 9, 12 and 18 months to measure length (using a Shorr board), weight (using a Seca 384 scale), MUAC, and administer a structured questionnaire. All monitors’ measurements were validated twice against a gold standard, and we calculated intra-observer and inter-observer technical error of measurement for length and other anthropometric outcomes.

We use mobile phones programmed using the CommCare platform for data collection. The monitoring team checks the number of interviews completed and type of data collected every week in order to promptly identify and address possible problems. The data are downloaded every two weeks and checked for errors using automated do files and manual inspections. Names of participants are collected but removed before data storage. Final datasets contain numerical identifiers for cluster individual participants. The data are stored and backed up every two weeks on a server.

### Blinding

Due to the nature of the intervention, the participants and intervention teams are not blinded to the allocation of clusters. While members of the monitoring team are not given information about the allocation of individual clusters during their training, they are likely to see some of the intervention workers (SPKs) and hear about them in the course of their monitoring activities, which makes complete blinding difficult. To minimise this effect, the intervention and monitoring teams meet on different days and do not discuss individual cases of mothers and children. The trial statistician will conduct interim and final analyses blind to allocation.

### Study outcomes

The primary outcome of the trial is children’s length-for-age Z scores of 2006 WHO Growth Standards at 18 months. Secondary outcomes are described in Additional file 1, along with details of their inclusion in the interim Data Monitoring Committee (DMC).

### Analysis plans

#### Interim analysis

We do not expect any adverse effects of the intervention but plan to carry out two interim analyses in 2014 and 2015, which will be reported to an independent DMC convened according to the DAMOCLES charter [45]. At these meetings, the DMC will examine data for the following:

- Recruitment and retention (i.e. the number of births registered in the study and followed up);
- Mean length for age z score and weight for height z scores at the most recent measurement for each child
- Two measures of intervention coverage: % of mothers visited by an SPK in the last three months; % of mothers who attended a women’s group meeting in the last three months;
- Two measures of monitoring quality: mean difference in days between expected and actual interview dates; average difference between first and second length measurement for most recent measurement).

The DMC members will look at these data by allocation arm independently, write down their immediate assessment, and then contribute to a general discussion with the research team. At the first interim analysis in 2014, the DMC observed a shortfall in the number of live births compared to what was expected in the sample size calculation. The DMC recommended extending the recruitment period until we reached the target sample size. This was achieved in February 2015.

#### Final analysis

Final analyses will be by intention-to-treat and include all mothers who were pregnant and had a live birth during the recruitment period (1st October 2013 – 10th February 2015) and their infants, regardless of whether they received the intervention or not. We will test for differences between intervention and control areas in the main outcomes of interest using linear regression for continuous outcomes and logistic regression for binary outcomes, adjusting for clustering using random effect models. We will examine levels of missing data for the primary and secondary outcomes, and plan to use multiple imputation if these exceed 10%.

For the final DMC, we plan to carry out three sub-group analyses. The first will examine differences in children’s LAZ at 18 months according to the intensity of their mothers’ exposure to group meetings (number of meetings attended), adjusting for maternal education, multi-dimensional poverty status, and tribal status. The second sub-group analysis will examine differences in growth outcomes between arms by wealth quintile to understand the equity impact of the intervention. The third sub-group analysis will examine differences in growth outcomes for boys and girls. The final analysis will be presented according to the CONSORT requirements for cluster randomised controlled trials [46,47]. Both the interim and final analyses will be conducted by the trial statistician (A Copas).

### Process evaluation

A process evaluation will document the design, implementation and mechanisms of the intervention to enable replication and scale up. Five main sources of data will be used: (a) project planning and monitoring documents; (b) forms to capture attendance, problems and strategies identified by the women’s groups; (c) SPK home visit diaries noting the three main issues discussed during visits; (d) around 20 semi-structured interviews (data will be collected until saturation) with mothers of children under-2 in the experimental arm and eight focus group discussions with small family groups, SPKs, *Anganwadi*s and ASHAs to document perceptions of the intervention and its effects; (e) field notes from intervention team members to be reviewed through monthly debriefings will document intervention events.

### Economic evaluation

We will conduct cost and cost-effectiveness analyses to guide policy decisions and inform any subsequent scale up. We will estimate the total and incremental costs of the intervention from a project, provider, household and societal perspective. The project costs of delivering the interventions will be collected prospectively from the project accounts using a standardised data capture tool designed for this purpose in Excel, and converted to economic costs. Costs incurred by health care providers (mainly MTCs in the districts) will be collected using trial monitoring data on the change in service use, combined with key informant interviews and available financial data on the average unit cost of delivering those services. Patient (household) costs will be collected prospectively during follow-up surveys. All costs will be adjusted for inflation using the Indian Consumer Price Index (CPI) and will be presented in Indian Rupees and 2016 International Dollars. Incremental cost-effectiveness will be measured in relation to the *status quo* alternative i.e. doing nothing in addition to the current ongoing activities. Incremental cost-effectiveness ratios (ICERs) will be calculated for the primary outcome measure (i.e. cases of stunting prevented) and selected secondary and summary measures including cases of wasting prevented, cases of infant mortality averted, life-years saved and DALYs averted. Sensitivity analyses will be carried out to assess the robustness of results.

### Ethical issues

The study received ethical approval from the research ethics committee of the Public Health Foundation of India (June 2013, TRC-IEC-163/13), an Independent Ethics Committee linked to Ekjut (May 2013), and University College London’s Research Ethics Committee (June 2013, reference 1881/002). We identified and sought to address five main ethical concerns.

#### 1. The need for cluster-level and individual-level informed consent for participation in the trial

We sought cluster-level consent from village leaders and village health, sanitation and nutrition committee members after explaining the study aims, intervention and data collection procedures. We also seek individual consent from mothers prior to each interview and measurement session; consent is in writing or by thumbprint at the first interview, and verbally for subsequent interviews.

#### 2. Identification of severely acutely malnourished children

For ethical reasons, severely acutely malnourished (SAM) children identified by the study monitoring team in both intervention and control arms are all referred to the *Anganwadi Sevika* for onward referral to the local MTC.

#### 3. Benefits to control areas

In addition to the referral of SAM children in both arms, we are implementing a capacity building intervention for local VHSNCs in both the trial intervention and control arms.

#### 4. Impact of the intervention on existing health services

The trial activities can have several repercussions, both positive and negative, on NRHM and ICDS services. First, we expect our work with VHSNCs to impact on the provision and uptake of local health and nutrition services, including demand for, and utilisation of, Malnutrition Treatment Centres. We will monitor process indicators for the VHSNC intervention by assessing members’ awareness of entitlements and service guarantees at the beginning and end of the intervention through a questionnaire. We will also measure changes in the utilization of MTCs. Secondly, it is possible that the presence of a second health and nutrition worker may cause tension with other community workers. It is therefore critical for the study team and SPKs to establish role and supervision boundaries with the districts’ ICDS and NRHM teams prior to the start of the study, and for the SPKs to establish a rapport with existing community health workers at the beginning of the intervention. This was done by engaging these workers in the selection of the SPKs and with an introductory meeting between SPKs and other workers at the outset.

#### 5. Use of a parallel trial design

Finally, the use of a parallel design for this study has ethical limitations: in areas with such high levels of stunting and where no group meetings or home visits are available to mothers and children, the equipoise of this study (i.e. genuine uncertainty over its benefits) can be questioned. However, we selected this design over a stepped-wedge approach due to concerns about the increased sample size that a stepped-wedge design might entail and the lengthy time frame required to implement a sufficient number of steps. We believe that operational research studies such as this would benefit from further methodological work on alternative approaches to parallel-design trials, including phased designs [48].

### Intervention sustainability and scalability

We developed the community strategy to be manageable for a single community-based worker within existing NRHM and ICDS systems. *Suposhan Karyakartas* have similar educational levels as *Anganwadi Sevikas*, the same stipend and the same supervisor to worker ratio (1:10). The trial results will be generalisable to rural areas of India with a high proportion of poor, Scheduled Tribe and Scheduled Caste communities. We estimate that these constitute at least 70% of ICDS’ 200 high-burden districts across India, or a population of over 170 million. We will seek to maximise generalisability and impact through three strategies. First, engagement with the health system will start from the village-level, as the SPK will aim to work in collaboration with existing frontline workers and the VHSNCs. Second, we will engage with ICDS and NRHM implementers at the onset, midpoint and end of the study to ensure the intervention’s compatibility with existing systems and maximise its scalability. Third, if the strategy improves the trial’s primary and/or secondary outcomes, we will use our cost-effectiveness estimates and process evaluation data, together with socio-demographic and health systems data from the 200 ICDS high-burden districts, to assess the scalability of the intervention through NRHM and ICDS systems, and to model the potential costs and impact of the intervention scaled up to all or parts of the high-burden districts.

### Trial timeline and status

The trial is planned for a duration of 46 months (1st April 2013 – 31st January 2017) and is currently recruiting participants.

## Additional files

Additional file 1:
**Supplementary Table 1.** Trial outcomes

## Abbreviations

ANM: Auxiliary Nurse Midwife
ASHA: Accredited Social Health Activist
AWW: Anganwadi Worker
DMC: Data monitoring committee
ICDS: Integrated Child Development Services
NGO: Non-government organization
NRHM: National Rural Health Mission
SD: Standard deviation
SPK: *Suposhan Karyakarta*
