# Supplementary material for: Participatory women’s groups and counselling through home visits to improve child growth in rural eastern India: protocol for a cluster randomised controlled trial
Source: BMC Public Health. 2015 Apr 15;15:384. doi: 10.1186/s12889-015-1655-z (PMC4410595; doi:10.1186/s12889-015-1655-z)
Supplement: Additional file 1: — Supplementary Table 1. Trial outcomes [file 12889_2015_1655_MOESM1_ESM.pdf]

Supplementary Table 1: Trial outcomes

Primary outcome

Mean length for age z score at 18 months

Secondary outcomes

Anthropometry
Mean weight for height z score at 18 months
Mean weight for age z score at 18 months
Mean MUAC z score at 18 months
% of children who are stunted at 18 months
% of children who are underweight at 18 months
% of children who are wasted at 18 months
Mean birth weight
Change in weight from birth to 18 months
Change in height from birth to 18 months
Mean length for age z score at 72h, 3, 6, 9 and 12 months
Mean weight for height z score at 72h, 3, 6, 9 and 12 months
Mean weight for age z score at 72h, 3, 6, 9 and 12 months
Mean MUAC z score at 6, 9 and 12 months
Mean maternal MUAC in third trimester of pregnancy
Mean maternal BMI 9 months after delivery

Health and nutrition in pregnancy
% mothers receiving 3+ ANC by qualified provider
% mothers receiving minimum IFA supplements and two TT injections
% mothers with self-reported symptoms of anaemia, vaginal bleeding or malaria
% mothers seeking care for any of the above
% mothers who delivered with a skilled attendant
% mothers and children who received one or most postnatal check-up(s)
n of months since previous birth
Mean age at marriage
Mean number of meals per day
% mothers with minimum dietary diversity

Infant and Young Child Feeding
% infants breastfed within one hour of birth
% infants exclusively breastfed until 6 months
% infants who started complementary foods at six months
% infants still breastfed at one year
% children given food from four or more groups at 6, 9, 12 and 18 months
% children given minimum meal frequency at 6,9, 12 and 18 months
% children given iron-rich foods at 6, 9, 12 and 18 months
% children given a source of protein at 6, 9, 12 and 18 months

Care-giving and care-seeking during illness
% children with diarrhea, cough, fever in past 2 weeks
% children receiving appropriate care during illness episode (fluid replacement for diarrhea and continued feeding for all illnesses)
% children for whom care was sought from appropriate provider
% children who received appropriate treatment from qualified provider

Infection control

% children who received BCG, OPV3, DTP3, measles and Hep. B
Vitamin A dose in last 6 m (children 6-18 m)
% of children who received deworming in the past 6 months
% mothers who keep soap in the household
% mothers who keep water container covered
% mothers reporting handwashing with soap before feeding a child
% mothers reporting handwashing with soap after helping a child to defecate
% mothers reporting handwashing with soap after defecation
% of mothers who report placing child feces in latrine or burying them

Mortality

Infant mortality rate (per 1000 livebirths)

Receipt of ICDS entitlements

% pregnant women who received Take Home Rations from AWW in previous month
% mothers who received THR for their child from AWW in previous month
% children weighed by AWW in previous month
% mothers who received nutritional counselling by AWW in previous month
% mothers who have received entitlement through Odisha's Mamta Scheme

Maternal mental health and decision-making

% mothers with K10 score > 16 (indicating moderate to severe psychosocial distress)
% mothers with a lot or complete control over decisions about their own healthcare
% mothers with a lot or complete control over decisions about major household purchases
% mothers with a lot or complete control over decisions about daily household purchases
% mothers who have money of their own and over which they have complete control over
